# Beneficial Effect of Systemic Allogeneic Adipose Derived Mesenchymal Cells on the Clinical, Inflammatory and Immunologic Status of a Patient With Recessive Dystrophic Epidermolysis Bullosa: A Case Report

**DOI:** 10.3389/fmed.2020.576558

**Published:** 2020-11-26

**Authors:** Rocío Maseda, Lucía Martínez-Santamaría, Rosa Sacedón, Nora Butta, María del Carmen de Arriba, Sara García-Barcenilla, Marta García, Nuria Illera, Isabel Pérez-Conde, Marta Carretero, Eva Jiménez, Gustavo Melen, Alberto M. Borobia, Víctor Jiménez-Yuste, Ángeles Vicente, Marcela del Río, Raúl de Lucas, María José Escámez

**Affiliations:** ^1^Department of Dermatology, La Paz University Hospital, Madrid, Spain; ^2^Department of Bioengineering, Carlos III University (UC3M), Madrid, Spain; ^3^Rare Diseases Networking Biomedical Research Centre (CIBERER) U714, Madrid, Spain; ^4^Regenerative Medicine and Tissue Engineering Group, Health Research Institute Foundation of the Jiménez Díaz Foundation, Madrid, Spain; ^5^Centre for Energy, Environment and Technology Research (CIEMAT), Madrid, Spain; ^6^Department of Cell Biology, Faculty of Medicine, Complutense University, Madrid, Spain; ^7^Hematology Unit, La Paz University Hospital-IdiPAZ, Madrid, Spain; ^8^Cell & Gene Therapies Laboratory, Niño Jesus University Hospital, Madrid, Spain; ^9^Clinical Pharmacology Department, School of Medicine, La Paz University Hospital, IdiPAZ, Autonomous University of Madrid, Madrid, Spain

**Keywords:** recessive dystrophic epidermolysis bullosa (RDEB), systemic cell therapy, mesenchymal stromal cells (MSC), inflammation, case report, adipose derived MSC (ADMSC)

## Abstract

Recessive dystrophic epidermolysis bullosa (RDEB) is an incurable inherited mucocutaneous fragility disorder characterized by recurrent blisters, erosions, and wounds. Continuous blistering triggers overlapping cycles of never-ending healing and scarring commonly evolving to chronic systemic inflammation and fibrosis. The systemic treatment with allogeneic mesenchymal cells (MSC) from bone marrow has previously shown benefits in RDEB. MSC from adipose tissue (ADMSC) are easier to isolate. This is the first report on the use of systemic allogeneic ADMSC, correlating the clinical, inflammatory, and immunologic outcomes in RDEB indicating long-lasting benefits. We present the case of an RDEB patient harboring heterozygous biallelic *COL7A1* gene mutations and with a diminished expression of C7. The patient presented with long-lasting refractory and painful oral ulcers distressing her quality of life. Histamine receptor antagonists, opioid analgesics, proton-pump inhibitors, and low-dose tricyclic antidepressants barely improved gastric symptoms, pain, and pruritus. Concomitantly, allogeneic ADMSC were provided as three separate intravenous injections of 10^6^ cells/kg every 21 days. ADMSC treatment was well-tolerated. Improvements in wound healing, itch, pain and quality of life were observed, maximally at 6–9 months post-treatment, with the relief of symptoms still noticeable for up to 2 years. Remarkably, significant modifications in PBL participating in both the innate and adaptive responses, alongside regulation of levels of profibrotic factors, MCP-1/CCL2 and TGF-β, correlated with the health improvement. This treatment might represent an alternative for non-responding patients to conventional management. It seems critical to elucidate the paracrine modulation of the immune system by MSC for their rational use in regenerative/immunoregulatory therapies.

## Introduction

Recessive dystrophic epidermolysis bullosa (RDEB) is a mechano-bullous genodermatosis due to biallelic mutations in the *COL7A1* gene leading to a decrease in or complete absence of collagen type VII (C7) at the mucocutaneous surfaces and the extracellular matrix of multiple organs. As a result, alterations in the anchoring fibrils (AF) (1) cause fragility and lifelong blistering below the basement membrane which is the hallmark of the disease. Never-ending healing and progressive scarring continuously triggered by the generation of new blisters, lead to chronic wounds characterized by increased bacterial colonization, fibrosis, and inflammation, which eventually evolve as a systemic condition with an increased risk of developing cutaneous squamous cell carcinoma (2–10). Current RDEB treatments are palliative. Evidence-based therapeutic approaches are developing but still far from entering clinical practice (11). Meanwhile, knowledge on secondary events that influence the course of the disease has led to the development of symptom relief therapies. In fact, systemic administration of allogeneic bone marrow-derived mesenchymal stromal cells (BMMSC) has shown benefits in RDEB without severe adverse effects (12–14). It is generally accepted that the therapeutic mechanisms underlying MSC are mostly correlated to paracrine actions (15, 16) by regulation of the behavior of immune cells (17, 18). MSC from adipose tissue are easier to isolate and have similar therapeutic potentials than those from bone marrow (19, 20).

## Case Description

**Figure 1:**
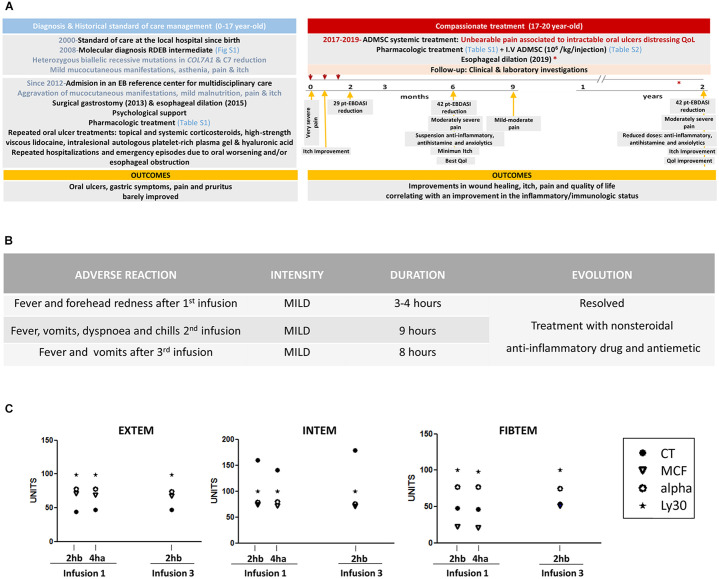
Case report timeline and assessment of the tolerability of systemically administered ADMSC **(A)** Temporal diagram representing major events, interventions and outcomes. Day 0 (first infusion): October 5, 2017, Day 21 (second infusion): October 26, 2017, Day 42 (third infusion): November 16, 2017, 3 months: November 30, 2017, 4 months: January 11, 2018, 6 months: April 9, 2018, 9 months: July 19, 2018; 1 year: October 4, 2018; 2 years: November 11, 2019. **(B)** Adverse reactions registered during the 12 h following all three infusions. **(C)** Hemostatic parameters measured just 2 h before (2hb) and 4 h after (4ha) the first infusion and 2 h before third infusion (2hb). Rotational thromboelastometry (ROTEM) parameters determined for EXTEM, INTEM, and FIBTEM tests are shown. EXTEM test, extrinsic pathway thromboelastometry; INTEM test, intrinsic pathway thromboelastometry; FIBTEM, fibrinogen thromboelastometry. Clotting time (CT, time from the start of measurement to the start of clotting, in seconds); alpha angle (tangent to the curve at 2 mm amplitude, in degrees, which reflects the rate of fibrin polymerization); maximum clot firmness (MCF, in mm, which reflects the maximum tensile strength of the clot); and lysis at 30 min (Ly30, in %; residual clot firmness 30 min after CT).

Our patient was a 17-year-old Caucasian woman with RDEB, born to term without remarkable findings during her mother's pregnancy. Her unrelated parents had an older healthy child. There was no family history of skin blistering disorders. Since birth, the patient developed cutaneous lesions, mucosal involvement, and onychodystrophy. Molecular diagnosis disclosed compound heterozygosity of the highly recurrent Spanish nonsense mutation p.G2177Wfs113^*^ (c.6527insC) (21) with a missense mutation p.G2434R (c.7300G>A) leading to the diagnosis of RDEB intermediate (22–24) and consistent with reduced expression of C7 (Appendix 1 and Supplementary Figure 1). In 2008, the patient presented with small cutaneous active erosions and blisters mostly located in friction areas, post-inflammatory hyperpigmentation, milia, atrophic scarring, 17 absent and 3 dystrophic nails as well as mild flexion contractures. Buccal manifestations and gastro-esophageal reflux were mild but already the main concern together with asthenia, pain, and itch. No history of significant seasonal worsening was registered. Since 2013, the patient -presenting with more generalized skin lesions, mild alopecia, digital contractures, and xerophthalmia- had been under routine multidisciplinary care at La Paz University Hospital. Skin care to prevent infections and dryness consisted of the alternating and/or combined use of standard dressings. Severe microstomia and ankyloglossia limited oral feeding, contributing to a mild nutritional compromise and dysphagia for solids, which was ameliorated by surgical gastrostomy and esophageal dilation. Ulcers in the oral mucosa, causing bleeding and unbearable pain, progressively worsened and failed to respond to a variety of repeated treatments (Figure 1A), causing frequent hospitalizations and emergency episodes. Depression, pain and intense generalized itch often precluded the patient from attending school and limited her social activity with distressing her quality of life. Alongside psychological support, a combination of opioid analgesics and low doses of tricyclic antidepressant and histamine antagonists (Supplementary Table 1) were administered on a long-term basis, but barely improved gastric symptoms, pain, and pruritus. Therefore, in the context of compassionate use, a concomitant systemic cell-based treatment was offered in 2017 (Figure 1A).

## Therapeutic Intervention: Systemic Cell-Based Therapy for Symptom Relief

The procurement and manufacture of allogeneic ADMSC were performed according to currently applicable regulations (Appendix 1, Supplementary Table 2) and as previously described (25). After authorization by the Spanish Agency of Medicines and Health Products (AEMPS), three separate intravenous injections of 10^6^ ADMSC per kg of body weight, were administered to the patient every 21 days. Prior to infusion, 1 g paracetamol and 5 mg dexchlorpheniramine were provided. A 50 ml MSC-suspension was infused via a peripheral cannula and the patient remained under observation for at least 4 h. Vital signs were checked before and after infusion.

## Follow-Up and Outcomes

Physical examination and analytical tests recommended for EB (26) were performed on the infusion days (days 0, 21, and 42) and 2, 3, 6, and 9 months, and 1 and 2 years after the first infusion. Changes in medication were recorded (Supplementary Table 1). Since no evidences support that allogeneic MSC lead to an increase in C7 in previous clinical trials (12–14), skin biopsies to check this matter, were not programmed. Rotational thromboelastometry (ROTEM®) and fibrinogen assay (Clauss Method) were performed (27). The therapeutic effect was clinimetrically assessed by the Birmingham Epidermolysis Bullosa Severity Score (BEBSS) (28), the Epidermolysis Bullosa Disease Activity and Scarring Index (EBDASI) (29), Leuven Itch Scale (LIS) (30, 31), Visual Analog Scale (VAS pain) (32) and quality of life questionnaire (EuroQol-5D) (33). Circulating levels of non-specific positive and negative acute phase reactants and inflammatory cytokines (ELISA) were assessed. Immune status was determined by monitoring peripheral blood leukocyte (PBL) populations by flow cytometric immunophenotyping. Outcome assessment is further described in Appendix 1.

The systemic administration of ADMSC was well-tolerated without serious adverse events (Supplementary Tables 3, 4). Findings in hepatic and renal functions were not clinically relevant. Common mild infusion-related adverse reactions were in remission within a few hours by the administration of ibuprofen and metoclopramide (Figure 1B). Hemostatic and ROTEM parameters such us platelet count (253 × 10^3^/μl), coagulation time (11.5 seg), PT, aPTT, CT-EXTEM, MCF-EXTEM, and CT-INTEM were within the normal range and not affected by the infusion (Figure 1C). Of note, MCF-FIBTEM was increased at the third infusion (51 mm, normal range: 9–25). History or signs of malignancy were absent at baseline and during the 2 years follow-up.

**Figure 2:**
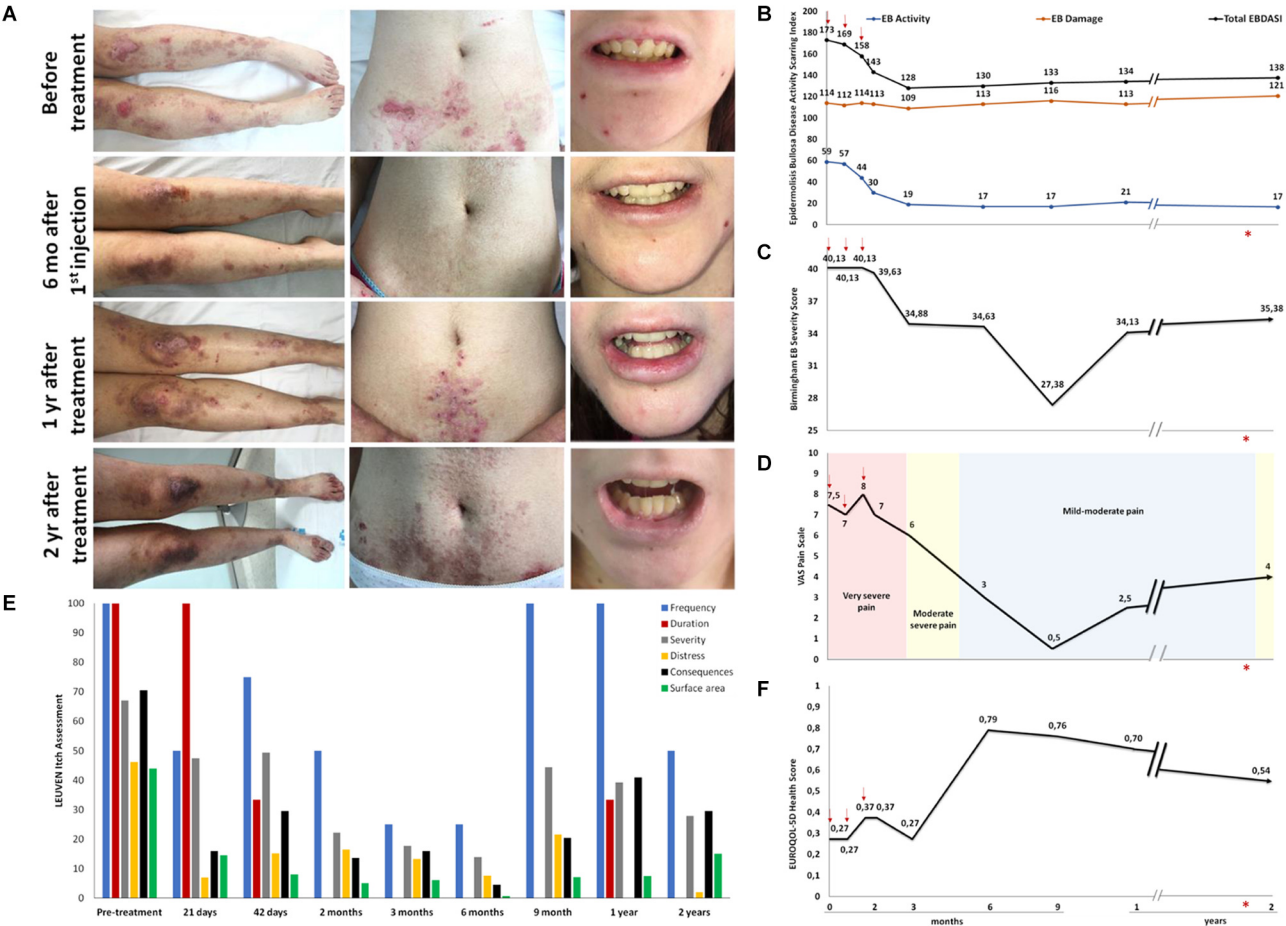
Assessment of the therapeutic effect of systemically administered ADMSC **(A)** Photographic images of legs, abdomen and mouth before and at various time points after the treatment showing a cutaneous improvement clinimetrically quantified by two specific severity index/scores. **(B)** EBDASI and **(C)** BEBSS. Mucocutaneous improvement after the treatment parallels a decrease in the scores for **(D)** pain (VAS) and **(E)** itch (LIS), as well as an improvement in **(F)** patient's quality of life (QoL-5D). Red arrows indicate first, second and third infusion. *: esophageal dilation.

After allogeneic ADMSC systemic administration, therapeutic benefits improving the patient's quality of life (QoL) were observed. Specifically, a progressive amelioration in the percentage of body surface covered by lesions dropped from 23 to 12% at 6 months after the first injection (Figure 2A). The oral cavity was not examined because of unbearable pain, which was progressively mitigated after the treatment. The patient also reported less difficulty opening her mouth. BEBSS (28) and EBDASI (29), which measure changes in the mucocutaneous pathology, impressively reduced at 3 months, reaching minimal values at 6 and 9 months post-treatment, respectively (Figures 2B,C). While BEBSS scores increased gradually (remaining below the baseline at any time), EBDASI remained at minimal values up to 2 years. In both cases, sub-scores for affected skin area, mucous membranes and other epithelialized surfaces including larynx and esophagus were diminished (Supplementary Tables 5, 6). EBDASI damage score was scarcely affected. On a VAS-scale (32), pain intensity decreased immediately after the third infusion reaching the minimum level at 9 months (Figure 2D). According to the LIS questionnaire (30, 31), the patient experienced an improvement in all aspects of itch (except duration) 21 days after the first infusion, reaching minimum values after 6 months (Figure 2E). At this time, anti-inflammatory, antihistamine and anxiolytics were suspended and doses of antacids and analgesics were reduced (Supplementary Table 1). Two years after ADMSC infusion, the pharmacologic treatment was still at lower doses than at baseline. Regarding EuroQol-5D (33), the negative impact of the disease on patient's mobility, self-care and usual activity alongside maximum pain gradually improved, with the best QoL at 6 months after the treatment (Figure 2F). Two years post-treatment, the patient still considered her QoL as being better than at baseline and reported that she was able to regularly attend school and to enjoy social activity.

**Figure 3:**
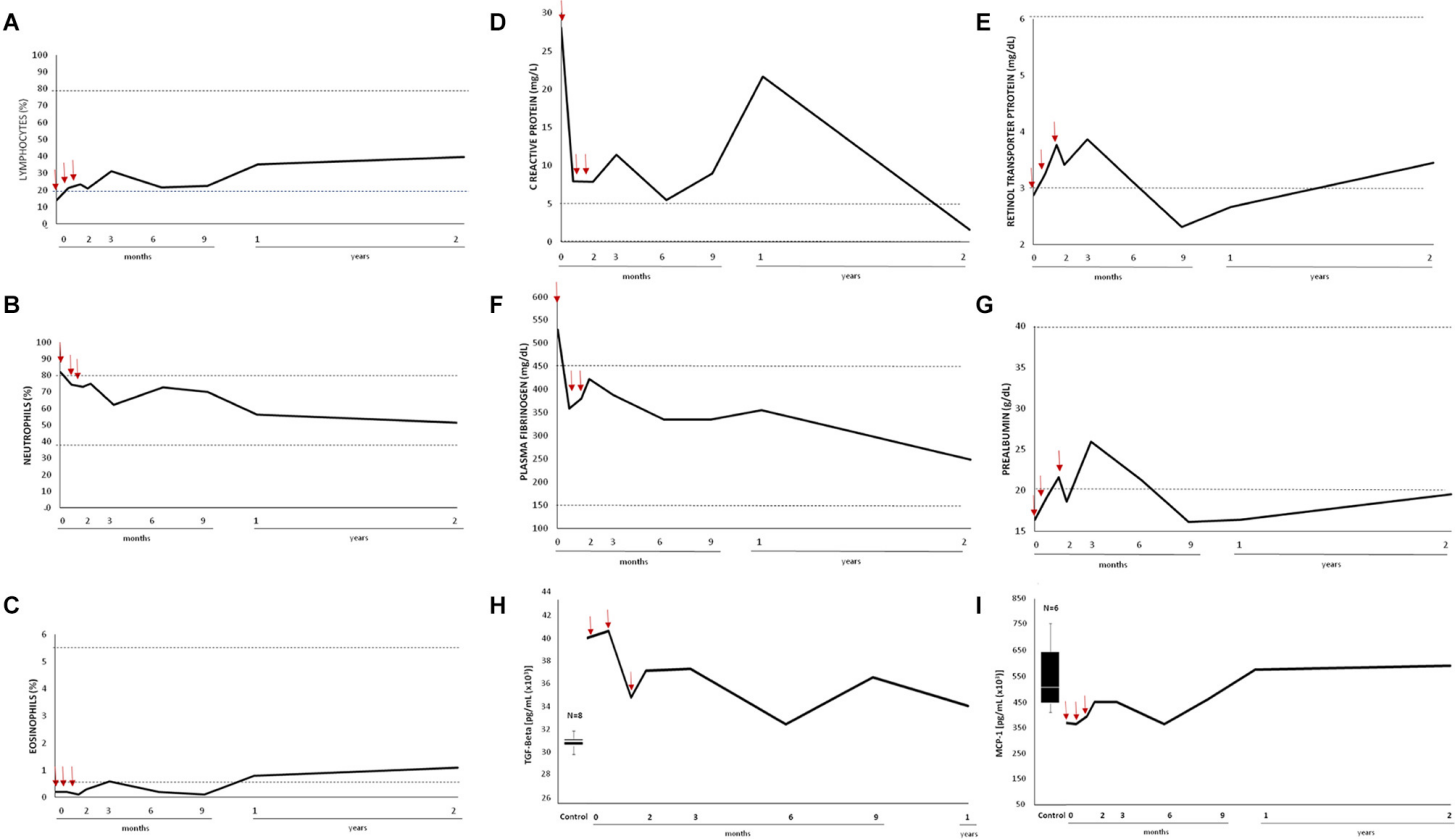
Impact of systemically administered ADMSC on non-specific inflammatory markers. White blood cell count represented as percentage of **(A)** lymphocytes **(B)** neutrophils, and **(C)** eosinophils. Circulating levels of **(D,E)** positive and **(F,G)** negative acute phase reactants. Dashed lines represent the reference rank in age-matched general population. Circulating levels of **(H)** total TGF-β and **(I)** MCP-1/CCL2 in the control group (Box plot) and the patient (black curve). Box plot representing the statistical median (white line), interquartiles (IQR; black box), and the lowest and highest data points (Tukey whiskers black lines) calculated from nine control individuals (three males and six females, aged from 24–63). The number of control individuals, after eliminating non-representative outliers, is indicated in every case (*n*). Red arrows indicate first, second, and third infusion.

The patient's white blood cell count was within normal limits but the relative proportion of some populations was abnormal at baseline (Supplementary Table 4). Low and high proportions of lymphocytes and neutrophils, respectively, were normalized after the first infusion; low eosinophil percentage reached normal values exclusively after the third infusion (Figures 3A–C). Amelioration in the mucocutaneous disease was accompanied by an improvement in nutritional and inflammatory markers (Supplementary Table 4 and Figures 3D–G), which were moderately abnormal at baseline. Decreased levels of prealbumin and 25-hydroxy-vitamin D, indicative of mild malnutrition, normalized after the second infusion for up to 6 months, along with a 3 kg weight gain (Figure 3G and Supplementary Tables 3, 4). In parallel, a substantial modulation in the patient's abnormal circulating levels of acute phase reactants occurred (Figure 3B). Remarkably, levels of C reactive protein (CRP) sharply decreased after the first infusion. They were close to normal after 3 months and within control values 2 years after the first infusion, although transiently peaked at 1 year point when the patient had an episode of pharyngitis (Figure 3D). Increased levels of fibrinogen normalized after the second infusion for up to 2 years (Figure 3F).

Circulating levels of serum cytokines (Supplementary Figure 3) were determined. The median was analyzed as the central tendency measure (34) (Appendix 1). An asymmetric and large dispersion in the interquartile range (IQR) was observed for most cytokines in the control sample (*n* = 9). In our patient, levels of IL6, IL1b, fractalkine, IL4, IL13 (into IQR), sCD40L, TNF-α, IFN-γ, IL2, IL15, and IL17A (into Q1) and VEGF (into Q3) can be considered normal and did not undergo relevant changes after the treatment. Leveling out of the maximum and minimum range was found under the median in the case of MCP-1/CCL2 and over the median for IL10 and TGF-β. Up-regulation of MCP-1/CCL2 after the first infusion was followed by a down-regulation of TGF-β after the third infusion (Figures 3H,I) whilst IL10 levels were mostly steady (data not shown).

**Figure 4:**
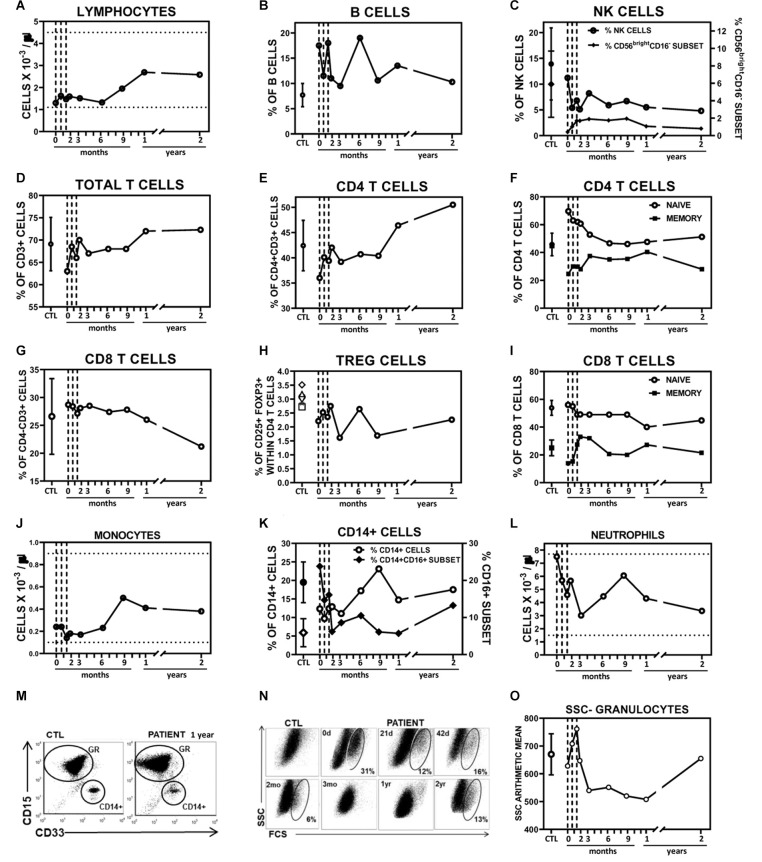
Impact of systemically administered ADMSC on peripheral blood populations. **(A–I)** Circulating lymphocyte populations. **(A)** Absolute numbers of lymphocytes (blood count tests). **(B–I)** Percentages of various subpopulations, within lymphocyte-gated cells, in patient and healthy control donors (CTL, mean ± SD; *n* = 6–7), analyzed by flow cytometry. **(B)** Percentages of B cells (CD19^+^) **(C)** Percentages of NK cells (CD56^+^CD3^−^, open circles, left axis) and CD56^bright^ CD16^−^ subset within total NK cells (filled diamonds, right axis). **(D)** Percentages of total (CD3^+^) T cells, **(E)** CD4 (CD4^+^CD3^+^), **(G)** CD8 (CD4^−^CD3^+^) T subpopulations and **(H)** TREG cells (CD25^+^Foxp3^+^) within CD4 T cells. Percentages of naïve (CD45RA^+^CD45RO^−^) and memory (CD45RA^−^CD45RO^+^) cells within CD4 **(F)** and CD8 **(I)** T cell subpopulations. **(J–O)** Circulating myeloid populations. Absolute numbers of monocytes **(J)** and neutrophils **(L)** obtained in patient blood count tests. **(K)** Percentages of CD14^+^ cells within the myeloid gate (open circles, left axis) and CD16^+^ subset within CD14^+^ cells (filled diamonds, right axis) found in patient and healthy control donors (CTL, mean ± SD; *n* = 6), analyzed by flow cytometry. **(M)** Representative dot plots showing CD15 vs. CD33 expression on granulocyte (GR) and CD14^+^ gated cells of a control donor and patient samples as indicated. **(N)** Dot plots show forward (FSC) vs. size (SSC) scatter of granulocyte-gated population obtained by flow cytometry analysis. d, days after first infusion; mo, months after first infusion; yr, before (pre-treatment) and after (post-treatment) first infusion. **(O)** The line chart represents the SSC arithmetic means of granulocyte population in the patient and healthy control donors (CTL, mean ± SD; *n* = 7). Vertical grids indicate days of MSC infusion. Horizontal grids indicate ranges considered clinically normal.

The impact of systemically administered ADMSC on the patient's PBL populations was monitored (Figure 4 and Supplementary Tables 7, 8). Absolute numbers of lymphocytes were low but within the reference range up to 6 months after treatment when a gradual increase took place (Figure 4A). Hence, 1 year after the first injection the number of lymphocytes doubled the baseline value. The proportion of B cells was higher than the control range but changes after the treatment might not be clinically relevant (Figure 4B). NK cells, playing a major role in the host rejection of both tumors and virally infected cells, can be subdivided into two major subsets. The CD56^dim^CD16^+^NK subset mediates cytotoxicity and antibody-dependent cellular cytotoxicity whilst CD56^bright^CD16^−^ subpopulation primarily secretes immunoregulatory cytokines crucial to coordinating innate and adaptive immune responses (35). The proportion of total NK cells, normal at baseline, dropped to half after the second infusion for up to 1 year (Figure 4C) whilst the dramatically reduced proportion of the CD56^bright^ NK subset slightly increased after the treatment. Certainly, T cells were the lymphocyte population that showed the most important changes after ADMSC infusion (Figure 4D). The proportion of total CD3^+^ T cells, normal at all the time points in the study, progressively increased after the first infusion, largely because of the raise in the CD4^+^ T cell subpopulation (Figure 4E). Although MSC have been repeatedly described as potent inducers of immunotolerance (36), this effect was not reflected in an increase in FOXP3^+^CD25^+^ CD4 T cells in our patient (Figure 4H). Nevertheless, we observed an important transitory recovery of the markedly reduced proportion of memory T cells, which reached normal values after the third infusion up to 1 year (Figure 4F). Of note, CD8 memory cells doubled their proportion after ADMSC infusion to fourfold in absolute terms 1 year post-treatment (Figure 4I). Indeed, the suboptimal number of memory T cells may compromise long-term immunologic protection/surveillance (37). The main myeloid populations were also analyzed. The absolute numbers of monocytes were low but within the reference range at baseline and stable after 6 months post-treatment (Figure 4J). After this time-point, they increased both in absolute and percentage terms. The CD16^+^ subpopulation was increased in the patient before treatment, being 30% of monocytes whereas in healthy controls this was 4.5–15%. From the first ADMSC infusion, this proportion decreased and was within the control range (Figure 4K). Interestingly, the remarkably low level of CD33 (Singlec3) expression in our patient's monocytes and granulocytes, an inhibitory receptor used as a typical myeloid marker whose involvement in disease is not yet completely understood (38) was not modified after the treatment (Figure 4M). We also found important changes in granulocytes after ADMSC treatment. At baseline, the absolute number of neutrophils, although within the control range, was relatively high (Figure 4L). At this point, we also found an anomalous subpopulation with a larger size and cellular complexity (SSC) (Figure 4N) compatible with immature granulocytes. This population of likely immature neutrophils decreased gradually, disappearing completely 6 months post-treatment (Figure 4N) when the overall improvement in the disease was maximal (Figure 2) and the numbers and SSC of circulating granulocytes reached the lower values (Figure 4O).

## Discussion

Herein we describe the first-in-human systemic treatment of RDEB with allogeneic MSC from adipose tissue. This treatment was aiming to provide a medical solution to a patient with an urgent need to improve her condition, thus the standard pharmacologic treatment was not suspended. Additionally, we have performed exploratory investigations of the therapeutic consequences of systemically administered ADMSC. The presence of new C7 deposition or AF formation, were not tested since allogeneic MSC in general did not lead to increase in C7 in previous clinical trials (12–14). The patient, a young woman with RDEB intermediate and a long-term poor QoL, had painful intraoral lesions refractory to treatments alongside extensive and constant itch. In RDEB, micronutrient and vitamin deficiencies of multifactorial origin, including low intake due to oral and esophageal damage causing pain of nociceptive and neuropathic origin, are common (39, 40). As a result, patients exhibit different grades of malnutrition, anemia and inflammation (40–42) *via* complex interrelationships not fully understood. Peripheral neuropathology associated with inflammation (43, 44) may be underlying the chronic itch and pain, which are the first and second highest disease burdens for patients living with RDEB, respectively (45–47).

The choice of treatment was primarily based on extensive experience in the safe use of allogeneic MSC gathered in more than 1,000 clinical trials (48), some of which for mitigating chronic inflammation associated with wound healing and skin diseases (48–50). Encouragingly, the intravenous administration of BMMSC from healthy donors was already shown to be safe and beneficial for symptom relief in RDEB, particularly in reducing itch (13, 14). We took into consideration that adipose tissue is a more accessible and abundant source for MSC isolation using a surgical procedure with low morbidity (19). Furthermore, BMMSC and ADMSC appear to have an identical immunomodulatory capacity when obtained from the same donor (51) but ADMSC might longer retain multipotency (20, 52).

The administration of three separate intravenous injections (10^6^ ADMSC/kg/infusion) every 21 days was well-tolerated without significant adverse reactions as previously reported (13, 14, 48). Additionally, thromboelastographic parameters reflected a preserved function of the patient's hemostatic system along the study. Consequently, it can be assumed that the risk of thrombosis was not increased in our patient, contrary to what has been reported in anecdotal cases (53). MCF-FIBTEM, by inhibiting platelet activity, provides clot tracing in which fibrinogen levels are the major determinant, but also depends on the availability of factor XIII (54) and on the participation of blood cell components (except platelets) and coagulation factors of the extrinsic pathway (55). Therefore, increased MCF-FIBTEM values are usually related to fibrinogen levels. We did not find this expected relationship, maybe because of the involvement of other factors such as the patient's inflammatory status (53). Nevertheless, EXTEM and INTEM parameters, related to both the extrinsic and intrinsic clotting cascade respectively, were within normal ranges. And finally, although malignancy has not been reported as a long-term adverse event of systemic MSC treatment (48), it may represent an issue in susceptible individuals with diseases prone to cancer development such as RDEB (10, 14). Yet, our patient did not have a history or signs of malignancy prior to ADMSC treatment, nor for the following 2 years.

After ADMSC systemic administration, the patient noticed an improvement in the cutaneous and oral lesions alongside pain and itch mitigation, as clinimetrically confirmed. Specifically, improvements in BEBSS (28), EBDASI (29), LIS (30, 31), VAS-pain (32) and EuroQol-5D (33) scores peaked at 6–9 months after the treatment. Reductions in EBDASI activity score >9 points can be interpreted as clinically significant (56). In our case, we reached a reduction of 29 points as early as 2 months after the first infusion and a maximum reduction of 42 points at 6 months and up to 2 years. Two years after ADMSC infusion, the patient's perception of a better QoL, less pain and itch correlated with reduced severity scores and lower doses of the pharmacologic treatment.

EB is associated with a wide range of manifestations in multiple organs (57, 58) in which autoimmunity and inflammation aggravate the clinical outcomes (59–62). The presence of skin autoantibodies was ruled out in our patient in a previous study (63). Specifically, IgG and IgA were not detected in the basement membrane zone, and serological tests were negative for BP180/Col17, BP230, LAD, C7, and laminin γ. Furthermore, in the serum of our patient -with moderate levels of defective C7, a medium BEBS score (40.13/100) and elevated levels of CRP at baseline- normal levels of most important proinflammatory cytokines including IL6 were found. This cytokine profile could be partly due to the concomitant pharmacologic treatment since dexamethasone and antihistamine inhibit the transcription of proinflammatory cytokines (64–66). Beyond and besides, a significant down-regulation of TGF-β and normalization of MCP-1/CCL2 levels correlated with an important decrease in CRP (and other inflammatory markers), which remained lower than at baseline until the end of the study. Those molecules are typically induced by skin damage to promote wound repair but their overexpression is related to chronic inflammation, fibrosis and cancer in RDEB (9, 67–69). MCP-1/CCL2 has also been involved in modulating the severity of the disease (70, 71). Thus, targeting the TGF-β pathway might be a promising approach to symptom relief in RDEB and anti-fibrosis (3, 72), and is currently being explored in a phase I/II trial (REFLECT; EudraCT:2015-003670-32). Elevated CRP, one of the most common markers for inflammation and poor prognosis in fibrotic diseases (73, 74), may play an active role in those processes through TLR4/IRF3/NF-kappaB (75) and TLR4/NF-κB/TGF-β (76) pathways.

An intrinsic innate immune dysfunction in the secondary lymphoid organs directly associated with the lack of C7 in RDEB patients has been described (5). Our patient showed important alterations in PBL at baseline that affected both myeloid and lymphoid populations. Remarkably, the high percentage of circulating neutrophils and their cellular characteristics suggest their mobilization in an immature state (band cells) from bone marrow. However, the number of lymphocytes was low, being CD4 and CD8 memory T cells and CD16^−^CD56^bright^ NK cells the most affected populations. These alterations might reflect the patient's chronic inflammatory condition along with a compromised capacity to respond to new infections. Distribution of PBL populations is influenced by multiple factors, mainly mobilization from bone marrow and recruitment into damaged tissues –here skin and mucosae– that secrete attractant chemokines. EB-affected skin is infiltrated with CD45^+^ CXCR2^+^ hematopoietic cells, myeloid (CD16b^+^, CD11b^+^ cells) and lymphoid cells (CD45RA^+^, CD45RO^+^) that are recruited by a myriad of chemokines detected in active blisters (77). The progressive recovery of normal circulating PBL after ADMSC infusion correlates with the amelioration in skin lesions, inflammation and itching as well as the general improvement in QoL. Local and systemic effects of ADMSC could improve wound repair (78) and the subsequent decrease in local chemotactic signals indirectly allowing the recovery of PBL populations.

In summary, this is the first report on the use of systemic allogeneic ADMSC, correlating the clinical, inflammatory and immunologic outcomes in RDEB indicating long-lasting benefits. We recorded a gradual improvement in the disease severity, pain and itch that correlated with a positive change in the patient's QoL over a 2-year period. Reduction of itching (preventing the generation of new skin lesions caused by scratching) and inflammation has been pointed as factors contributing to improve skin integrity and/or dermo-epidermal adhesion during months after allogeneic MSC in RDEB patients (14). Remarkably, the lowest levels of TGF-β, the lowest EBDASI and LIS scores and the best QoL were reached at 6 months post-treatment when corticosteroids and antihistamine administration were suspended and an important recovery of PBL was occurring. Importantly, the normalization of systemic markers of inflammation and nutrition was accompanied by weight gain. Deficiency in vitamin D, which also has immunomodulatory properties (79) was also ameliorated. The impact of sporadic improvements in the disease activity cannot be neglected. However, it is important to recall that oral ulcers were unresponsive and persistent over 5 years prior to treatment. Thus, systemic ADMSC might represent an alternative for non-responding patients to conventional management. Indeed, this is a single case report and this issue should be further investigated preferably in the context of clinical trials. Our results support the growing body of evidence pointing the therapeutic capacity of MSC as immune modulators to stimulate the host's repair abilities (16–18) rather than their own capacity for structural repair and replacement. Therefore, at some point the endogenous repair abilities will not be able to counteract the cumulative effect of the inevitable appearance of new blisters (since the primary defect of mucocutaneous fragility has not been corrected) and the consequent inflammation would likely be triggering the itch-scratch cycle and further skin damage. Thus, the impact of additional doses would be worthy to be tested in future clinical trials. The cost of repeated treatment every 6 months with MSC, which can be performed in a two-hour visit to the hospital, would be comparable to the standard of care for RDEB patients (14).

## Patient Perspective

From the patient's point of view, she felt clear-headed and more energized for personal, academic, social and family care activities after treatment. She was optimistic and in a good mood appreciating the possibility of brushing her teeth and wearing contact lenses, earrings and high-heeled shoes. The patient and her mother still noticed oral and skin improvement 2 years after treatment, including food intake and a reduction in the number of dressings (only necessary to cover elbows). The patient and her family think that taking the treatment was a good decision.

## Data Availability Statement

The datasets presented in this study can be found in online repositories. The names of the repository/repositories and accession number(s) can be found at: https://www.ncbi.nlm.nih.gov/, PMID: 20184583.

## Ethics Statement

Individualized access to medicines in special situations (Royal Decree 1015/2009 of June 19) was authorized by the Spanish Agency of Medicines and Health Products (AEMPS). Written, signed and dated informed consents were obtained from the parents and the mature minor patient for the publication of any potentially identifiable images or data included in this article.

## Author Contributions

RM and LM-S contributed equally. MR, RL, and MJE conceived and designed the study and ensured funds for the treatment and the study. MJE directed the study, drafted, and revised all manuscript versions. RM, RL, IP-C, and SG-B were the team of physicians and nurses undertaking the treatment and sampling. LM-S and NI processed and managed biologic samples. RM and LM-S contributed to datasheet design, acquired clinical data and pictures. RS, EJ, and ÁV designed, performed, and interpreted PBL tests and wrote related sections. MCA contributed to the analysis of the results and compiled figures and graphs. NB and VJ-Y designed, performed, and interpreted the thromboelastographic and cytokine investigations and wrote related sections. MG and NI performed molecular diagnostics and provided related figures. MC and AMB contributed to the analysis of the results. GM was in charge of the production of the cell therapy medicine and wrote related sections of the manuscript. All authors contributed to the manuscript's revision, read, and approved the submitted version.

## Conflict of Interest

The authors declare that the research was conducted in the absence of any commercial or financial relationships that could be construed as a potential conflict of interest.
