# Supplementary material for: Beneficial Effect of Systemic Allogeneic Adipose Derived Mesenchymal Cells on the Clinical, Inflammatory and Immunologic Status of a Patient With Recessive Dystrophic Epidermolysis Bullosa: A Case Report
Source: Front Med (Lausanne). 2020 Nov 26;7:576558. doi: 10.3389/fmed.2020.576558 (PMC7726418; doi:10.3389/fmed.2020.576558)
Supplement: Supplementary file 1 [file Data_Sheet_1.PDF]

Supplementary Material

1. Appendix S1

1.1. Manufacture of expanded human allogeneic adipose-derived mesenchymal cells (ADMSC) for intravenous injection

1.2. Clinical and laboratory investigations

1.2.1. Physical examination

1.2.2. Analytical test

1.2.3. Hemostasis investigations and fibrinogen determination (Clauss Method)

1.2.4. Determination of cytokines and statistics

1.2.5. Analysis of peripheral blood leucocyte (PBL) populations

2. Supplementary Figures

Figure S1- Molecular diagnosis of recessive dystrophic epidermolysis bullosa.

Figure S2- Clinical presentation of the patient at baseline.

Figure S3- Basal status of patient's serum cytokines

3. Supplementary Tables

Table S1- Pharmacologic regimens

Table S2- Investigational Medicinal Product (IMP)

Table S3- Weight and vital signs measurements

Table S4- Hematologic and biochemical parameters

Table S5- Summary of selected items scored by BEBSS

Table S6- Summary of selected items scored by EBDASI

Table S7- Fluorochrome-conjugated antibodies for flow cytometry analysis

Table S8- Flow Cytometer setup parameters

1 Appendix S1

1.1 Manufacture of expanded human allogeneic adipose-derived mesenchymal cells (ADMSC) for intravenous injection

The donation, procurement and donor testing followed Good Clinical Practices (directive 2004/23/EC, 2006/17/EC and 2006/86/EC). Allogeneic ADMSCs were obtained by subcutaneous adipose tissue liposuction from a 30-year-old healthy female donor, under informed consent. Manual tumescent liposuction using a microcannula connected to a 50 cm^3 syringe was performed at the Plastic surgery department of La Paz University Hospital, under local anesthesia and aseptic conditions.

Manufacturing of the final medicinal product was carried out according to advanced therapy medicinal product (ATMP) guidelines and followed Good Manufacturing Practice (GMP) regulations (Directive 2003/94/EC). MSCs from lipoaspirate were isolated, expanded and packaged by the Cell and Gene Therapies Laboratory at the Niño Jesus University Hospital as previously described (25). In summary, the suctioned fat was digested with 0.45% collagenase type I (Sigma, Saint Louis, MO) in phosphate-buffered saline (PBS, Gibco, Carlsbad, CA) under gentle agitation for 60 min at 37ºC, and centrifuged at 1,400 rpm for 5 min to obtain the stromal cell fraction. The pellet were re-suspended, passed through a nylon filter of 40 µm to remove connective tissues, and cultured at 37°C in 5% CO2 in Dulbecco's Modified Eagle's Medium (DMEM, Gibco, Carlsbad, CA) supplemented with glutamine (Gibco, Carlsbad, CA), 10% fetal bovine serum (gamma-irradiated FBS, Gibco, Carlsbad, CA) and 1% streptomycin/penicillin (P/S, Sygma, Saint Louis, MO). After 24-72 hours, culture medium was changed to fresh, and cell confluence was evaluated. When primary cells reached 75% confluence, they were trypsinized (TripLE Express, Life Technologies, Carlsbad, CA) and seeded at 2:1 dilution. The secondary culture was maintained until MSC sub-confluence was reached. The adherent MSCs were harvested for cell banking: counted and cryogenized with 90% fetal bovine serum and 10% DMSO, followed by a series of quality control evaluations. Samples for mycoplasma and sterility tests were taken during final conditioning and performed following European Pharmacopeia, mycoplasma, and in accordance with the EU directive 2006/17 (EUD 2006/17/EC). Immunophenotype of adherent cells isolated from the stromal fraction was assessed by flow cytometry, being positive (≥ 85%) for CD73, CD90, CD29 and negative (≥ 95% positive) CD45, CD14 or CD19. For further details, see Table S2.

1.2 Clinical and laboratory investigations

1.2.1 Molecular diagnostic

Recommended standard methods consisting of analysis of C7 expression on a skin biopsy and direct bidirectional sequencing of COL7A1 on genomic DNA (gDNA) were followed (21,23,24). Briefly, immunofluorescence analysis was performed in cryosections by using LH7.2 monoclonal antibody at a 1:100 dilution (Sigma, St Louis, MO, U.S.A.). gDNA was extracted from blood using a commercial kit (Qiagen, Venlo, the Netherlands). Promoter region and all 118 exons and their flanking intronic COL7A1 sequences were polymerase chain reaction (PCR) and PCR products were sequenced with ABI Prism 3730 (Applied Biosystems, Foster City, CA, U.S.A). Parental gDNAs were also analyzed to confirm mutations and inheritance.

1.2.2 Physical examination

On physical examination ear, nose, throat, eyes, skin, mucosa and lymph nodes as well as gastrointestinal system, respiratory, cardiovascular, genitourinary and musculoskeletal systems, infection signs, mood, and DMSO odor were assessed. Weight, height, body mass index, body temperature, blood pressure, pulse rate and oxygen saturation were recorded (Table S3).

1.2.3 Analytical tests

Standard analytical tests were performed at the central laboratory of La Paz University Hospital as recommended for EB patients (26). Briefly, fasting peripheral blood samples were harvested by manual venipuncture. Samples were assessed for full blood cell count, nutritional status including trace mineral elements, glycemia and lipid metabolism, anemia, coagulation profile, hepatic function (including liver enzymes), renal function profile (including circulating electrolyte levels) and bone metabolism, thyroid metabolism, inflammatory status and immunologic profile (Table S4). Inflammatory status was assessed based on the levels of non-specific positive and negative acute phase reactants and the levels of various inflammatory cytokines including IL6 and IL10 as detailed bellow.

1.2.4 Hemostasis tests and fibrinogen determination (Clauss Method)

Rotational thromboelastometry (ROTEM®) was performed, as previously described (27), on citrated whole blood allowed to rest at room temperature for 30 min before testing. Tissue factor-activated extrinsic pathway (EXTEM test) and a partial thromboplastin phospholipid plus ellagic acid-activated intrinsic pathway (INTEM test) were performed to assess the kinetics of clot formation. Clotting time (CT, time from the start of measurement to the start of clotting, in seconds); alpha angle (tangent to the curve at 2 mm amplitude, in degrees, which reflects fibrin polymerization rate); maximum clot firmness (MCF, in mm, reflecting the maximum tensile strength of the clot); and lysis at 30 min (Ly30, in %; residual clot firmness 30 minutes after CT) were recorded. To assess the contribution of platelets to the clot kinetics, a platelet-inhibited FIBTEM test was also performed. The derived fibrinogen test result is extrapolated when running a PT assay using an optical endpoint detection system (Werfen, Madrid, Spain).

1.2.5 Determination of cytokines and statistics

Serum was isolated from whole blood collected without anticoagulants and after 20 minutes at room followed by refrigerated centrifuging at 1500 ×g for 10 minutes. Interferon-gamma (IFN-γ), interleukin (IL)-1β, IL-2, IL-4, IL-6, IL-10, IL13, IL-15, IL-17, monocyte chemoattractant protein 1 (MCP-1/CCL2), soluble CD40 ligand (sCD40L), tumor necrosis factor alpha (TNF-α), vascular endothelial growth factor (VEGF) and fractalkine were measured in serum samples with MILLIPLEX MAP Human Cytokine/Chemokine Magnetic Bead Panel (HCYTOMAG-60K, Merck, Madrid, Spain). Transforming growth factor beta (TGF-β) was measured by ELISA (R&D Systems, Madrid, Spain). Serum samples were analyzed in our patient at baseline and at various time points after the treatment. Serum levels of nine volunteers (3 males and 5 females, aged in between 24 and 63) were used as a control population.

For each cytokine, medians and interquartile ranges (IQRs), between 25% (quartile 1, Q1) and 75% (quartile 3, Q3) of the sample size were calculated. Box plots show median, IQR and Tukey whiskers values (outliers at maximums and minimums). Weight from non-representative outliers was removed, to reduce the large and asymmetric dispersion observed in the levels of most of the cytokines in the control sample, by eliminating extreme data. Values that were 1.5 times above Q3 or below Q1, the difference between Q3 and Q1 were considered non-representative outliers (34).

1.2.6 Analysis of peripheral blood leucocytes (PBL) populations

PBL populations were analyzed by flow cytometry before and after the treatment. EDTA-anticoagulated whole blood samples, obtained after venipuncture, were stained immediately after withdrawal. Following FC receptor blocking (FcR blocking reagent, Miltenyi), samples were incubated with specific fluorochrome-conjugated (FITC, PE, PercP-Cy5.5 and APC) antibodies for cell surface markers (Table S7): CD3 (HIT3a), CD4 (OKT4), CD8 (RPA-T8), CD45RA (HI100), CD45RO (UCHT1) for T cells; CD19 (HIB19) for B cells; CD56 (AF12-7H3) for NK cells; CD14 (47-3D6) and CD16 (VEP13) for monocytes and CD15 (HI98) and CD33 (P67.6) for granulocytes and monocytes from ImmunoStep (Salamanca, Spain), BioLegend, Myltenyi and BD Biosciences (Madrid, Spain). Then, erythrocyte cell lysis was performed with BD PharmLyse™ (BD Biosciences) and cells were fixed using BD CellFIX buffer. To analyze the FOXP3-CD25 CD4 T cell TREG subpopulation, peripheral blood mononuclear cells (PBMC) were isolated using a ficoll-based gradient (Rafer), FcRs were blocked and cell suspensions were stained for cell surface markers CD4, CD3 and CD25 (BC96, Biolegend). Intranuclear FOXP3 detection was performed using PCH101 antibody (eBiosciences, Madrid, Spain) and following eBioscience™ Foxp3 / Transcription Factor Staining Buffer Set-recommended protocol. All analyses were conducted in a FACSCalibur flow cytometer (BD Biosciences) from the Centre for Cytometry and Fluorescence Microscopy (Complutense University, Madrid, Spain). Flow Cytometer setup parameters are included in Table S8. Samples from healthy donors aged between 21 and 29 years were used as controls. Absolute numbers (cells/ml) of PB neutrophils, monocytes and lymphocytes were determined by conventional blood analysis.

2 SUPPLEMENTARY FIGURES

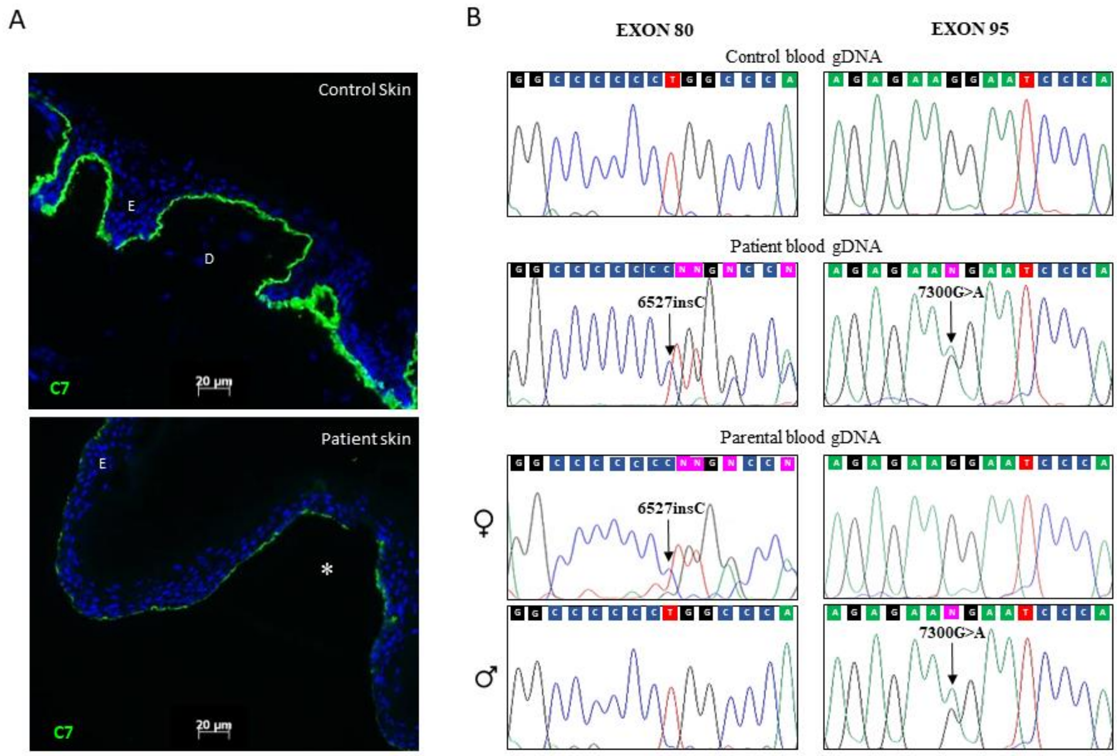

Figure S1- Molecular diagnosis of recessive dystrophic epidermolysis bullosa. (A) Microphotographic images of indirect immunofluorescence studies using a monoclonal antibody against the NC-1 domain of type VII collagen (Clone LH7.2) in control and patient skin. A decrease in C7 immunostaining at the dermoepidermal junction in the patient’s skin is evident when compared with the linear and bright labeling in control skin **(B)** Sanger sequencing chromatograms of genomic DNA (gDNA) extracted from peripheral blood demonstrating the presence of biallelic heterozygous mutations in the patient and in the corresponding progenitor. Control gDNA chromatograms are provided for comparison. E: Epidermis; D: Dermis; C7: Collagen 7; *: blister.

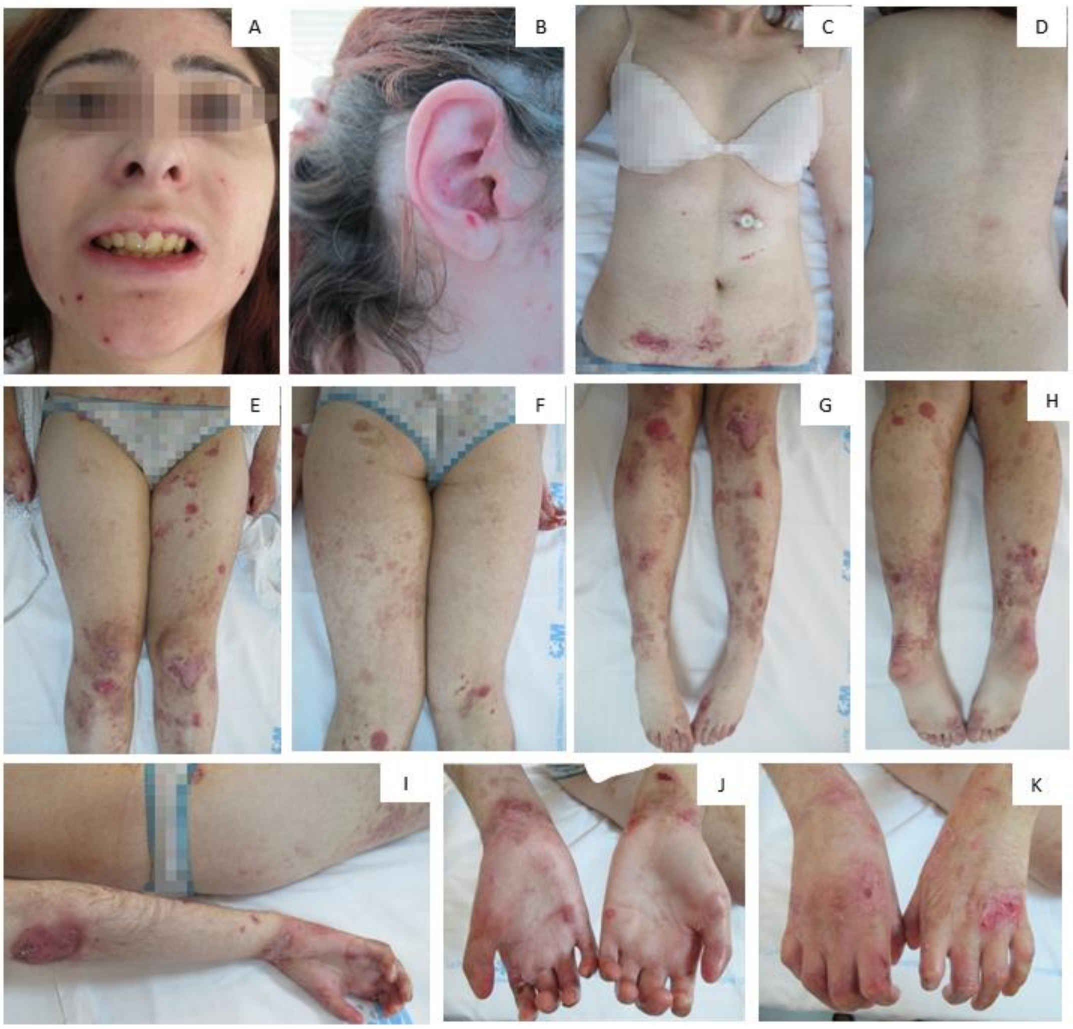

Figure S2- Clinical presentation of the patient at baseline. Before treatment at day 0, examination of the skin revealed that 23% of the body surface was covered with lesions and post-inflammatory hyperpigmentation macules. Photographic images of **(A)** face, **(B)** right ear, **(C)** anterior upper body, **(D)** back, **(E, F)** anterior and posterior upper legs, **(G, H)** anterior and posterior pretibial area and feet and **(I)** right elbow showing the distribution of erosions, atrophy and open wounds. **(J, K)** Images of the hands showing flexion contractures.

frontiers

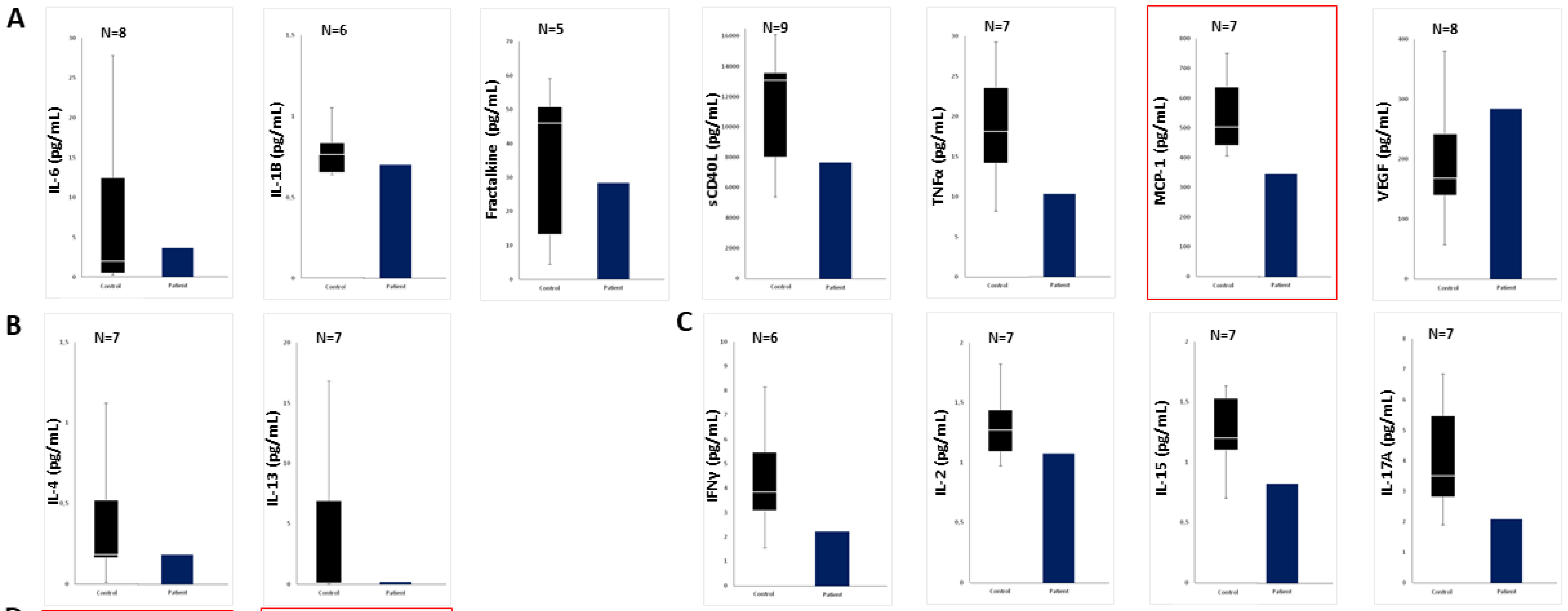

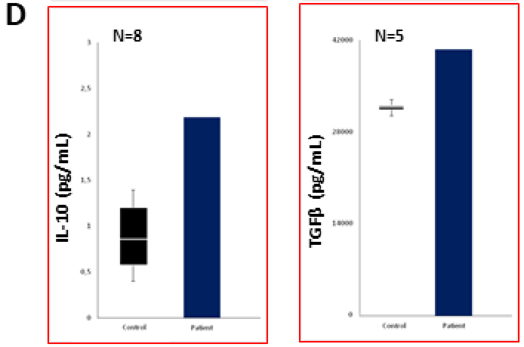

Figure S3- Patients' baseline serum cytokines (blue box). **(A)** Tissue damage cytokines **(B)** Th2 cytokines **(C)** Th1/Th17 lymphocyte subpopulation and **(D)** immunosuppressive and anti-inflammatory cytokines. Box plot representing the statistical median of the control group (white line), interquartile (IQR; black box), and the lowest and highest data points (Tukey whiskers gray lines) calculated from 9 individuals (3 males and 6 females, aged in between 24-63). A moderate or large dispersion of the IQR, mostly asymmetric, was observed in most of the cases, with outliers at maximums and minimums. The total number of control individuals, after eliminating non-representative outliers is indicated in every case (N). Levels considered anomalous, out of the maximum and minimum span, are framed in red.

3 SUPPLEMENTARY TABLES

Table S1- Pharmacologic regimens

ACTIVE PRINCIPLE	PRE-TREATMENT				POST-TREATMENT				
	-2.5 yr*	-2 yr*	-1 yr*	0 d	3 mo	6 mo	9 mo	1 yr	2 yr
**Dexamethasone 1 mg**	-	12 mg/day (2d)<br>8 mg/day (2d)<br>4 mg/day (2d)<br>4 mg/2day (4d)	Prednisone<br>30 mg/day	1 mg/day	1 mg/day	-	-	-	-
**21-Deflazacort 30 mg**			-	15 mg/day	7.5 mg/day	15 mg/day**	30 mg/day***	15 mg/day	7.5 mg/day
**Dexchlorpheniramine 2 mg**	6 mg/day	-	-	-	-	-	-	-	-
**Cetirizine hydrochloride 10 mg (Piperazine derivative)**			-	10 mg/day	10 mg/day	-	-	-	-
**Bilastine 20 mg (Piperidine derivative)**	-	-	-	-	-	-	20 mg /day	20 mg /day	-
**Ranitidine 150 mg**			300 mg/day	300 mg/day	300 mg/day	-	150 mg/day	300 mg/day	-
**Omeoprazole 20 mg**	-	-	-	20 mg /day	-	-	-	-	-
**Tramadol hydrochloride 100 mg/ml*** **		20-25 drops/8h	-	40 drops/8h	36 drops/8h	10-15 drops/8h	-	-	10 drops/day
**Morphine oral 2 mg/ml*** **	16 drops/4h		15 mg/2h	4 drops	4 drops	2-4 drops	-	4-6 drops<br>3-4 times/week	-
**Diazepam 5 mg**	-	5 mg/day***	5 mg/day	-	-	-	-	5 mg/day***	-
**Clonazepam 2,5 mg/ml**	-	-	-	-	-	-	-	-	4 drops/ night
**Dipotassium Clorazepate 5 mg**	-	-	-	5mg/day	-	-	-	-	-

d: days after first infusion; mo: months after first infusion; yr: years after first infusion.

* Medication from the emergency room registry after an urgent episode due to worsening of the oral mucosa and/or choking. ** During menstruation days. *** If needed.

Table S2- Investigational Medicinal Product (IMP)

SPECIFICATION

Infusion		First	Second	Third
Number		52 million cells	52 million cells	52 million cells
Viability		96.20%	99.30%	98.40%
Immunophenotype	CD90+	99.90%	99.10%	99.80%
	CD73+	100%	100%	99,90%
	CD29+	100%	100%	99,60%
	CD14+	0.00%	0.50%	0.10%
	CD19+	0.00%	0.00%	0.00%
	CD45+	0.00%	0.10%	0.10%

FORMULATION

Component	Adipose derived-MSC	0.9 % saline	2% human albumine
Quality	Good Manufacture Practice	Registered product	Registered product

Table S3- Weight and vital signs measurements

Parameter	Units	PRE-TREATMENT	POST-TREATMENT							
		0 d	21 d	42 d	2 mo	3 mo	6 mo	9 mo	1 yr	2 yr
Weight	Kg	52.5	-	-	54	51	55.4	55	55	51
Blood pressure	mmHg	109/61	108/59	110/67	114/64	114/66	115/67	113/69	114/66	97/59
Heart Rate	bpm	111	108	97	113	115	108	110	106	86
Temperature	°C	36.6	36.2	35.6	37.2	36.7	36.3	36.3	36.4	36.5
Respiratory Rate	bpm	21	16	15	21	15	16	20	16	12
O_2 Saturation	%	97	98	96	97	98	97	100	98	100

d: days after first infusion; mo: months after first infusion; yr: years after first infusion.

frontiers

Table S4- Hematologic and biochemical parameters

		REFERENCE RANK	PRE-TREATMENT			POST-TREATMENT							
			-2.5 yr	- 1.5 yr	0 days	21 days	42 days	2 mo	3 mo	6 mo	9 mo	1 yr	2 yr
INFLAMMATORY STATUS	WHITE CELL COUNT<br>Lymphocytes<br>Monocytes<br>Neutrophils<br>Eosinophils<br>Basophils	4.20–11.40 x10³ cells/mL<br>1.20-5.0 % (20-47 x10³ cells/mL)<br>0.10-0.95 % (1.5-9 x10³ cells/mL)<br>1.7-7.9 % (36-77x10³ cells/mL)<br>0.02-0.65 % (0.5-5.5 x10³ cells/mL)<br>0-0.2 % (0-1.8 x10³ cells/mL)	6.72<br>2.6 (38.8)<br>0.29 (4.3)<br>3.63 (54)<br>0.04 (0.6)<br>0.02 (0.3)	6.8<br>1.5 (25.4)<br>0.32 (5.2)<br>4 (65.6)<br>0.11 (1.7)<br>0.02 (0.4)	9.12<br>1.30 (14.2)<br>0.24 (2.6)<br>7.50 (82.2)<br>0.02 (0.2)<br>0.02 (0.2)	7.65<br>1.62 (21.2)<br>0.24 (3.1)<br>5.69 (74.4)<br>0.02 (0.2)<br>0.01 (0.1)	6.26<br>1.47 (23.5)<br>0.14 (2.2)<br>4.58 (73.1)<br>0.01 (0.2)<br>0.01 (0.1)	7.54<br>1.59 (21)<br>0.18 (2.4)<br>5.76 (75)<br>0.02 (0.3)<br>0.02 (0.3)	4.83<br>1.51 (31.2)<br>0.17 (3.5)<br>3.01 (62.3)<br>0.03 (0.6)<br>0.02 (0.5)	6.13<br>1.32 (21.6)<br>0.23 (3.7)<br>4.47 (72.9)<br>0.01 (0.2)<br>0.01 (0.2)	8.65<br>1.95 (22.6)<br>0.5 (5.8)<br>6.06 (70.1)<br>0.01 (0.1)<br>0.02 (0.2)	7.67<br>2.69 (35.1)<br>0.41 (5.4)<br>4.31 (56.2)<br>0.06 (0.8)<br>0.03 (0.4)	6.52<br>2.58 (39.6)<br>0.38 (5.8)<br>3.36 (51.5)<br>0.07 (1.1)<br>0.03 (0.4)
	C REACTIVE PROT.	0-5 mg/L	ND	23.06	28.05	7.23	ND	7.85	11.44	5.51	8.95	21.69	1.6
	PLASMA FIBRINOGEN	150-450 mg/dL	ND	662	529	359	381	423	389	335	ND	356	249
ANEMIA	Hematies	3.9-5.15 x 10⁶/mL	4.57	4.51	4	4.11	4.30	4.08	4.31	4.28	4.52	4.45	4.4
	Hemoglobin	12.0-15.4 g/dL	13.3	12.9	11.8	12.2	12.7	12.1	12.8	12.8	13.0	12.2	12.4
	Hematocrit	35.5-45.0 %	40.6	39.1	36.9	37.6	39.7	37.7	39.8	39.3	39.7	39.8	39.1
	Iron	50-170 ug/dl	86	101	68	136	78	104	70	90	12	34	105
	Transferrin sat. index	250-380 mg/dL (15-50%)	304 (22)	ND	259 (19)	ND	ND	ND	ND	ND	ND	264 (9)	ND
NUTRITION	Prealbumin,<br>Retinol transp. Prot.<br>Vitamin D	20-40 mg/dL<br>3-6 mg/dL<br>30-100 ng/dL	ND<br>ND<br>ND	19.70<br>3.43<br>21	16.40<br>2.88<br>25	19.2<br>3.26<br>31	21.6<br>3.77<br>27	18.6<br>3.41<br>30	25.9<br>3.87<br>30	21.2<br>3.05<br>36	16.1<br>2.31<br>21	16.4<br>2.67<br>17	19.5<br>3.4<br>28
	Vitamin A	0.30-0.70 mg/mL	ND	0.31	0.39	0.44	ND	0.42	ND	ND	ND	0.28	0.38

d: days after first infusion; mo: months after first infusion; yr: years before (pre-treatment) and after first infusion (post-treatment).
Abnormal values are represented in red and normalized values after the treatment in green

Renal parameters at baseline: creatinine: 0.54 mg/dL, glomerular filtration rate > 90 ml/min/1.73 m^2 body surface area, urea: 30 mg/dL, urate: 4.1 mg/dL, sodium: 4.1 mg/dL, potassium: 140 mmol/L, chloride: 103 mmol/L and total protein: 7.4 g/dL.. **Hepatic parameters at baseline:** aspartate transaminase, AST: 17 UI/L, alanine transaminase (ALT): 15 UI/L, alkaline phosphatase (ALP): 59 UI/L and 5'-nucleotidase (GGT): 15 UI/L. **Nutritional parameters at baseline:** serum levels for albumin (4 g/dL), trace mineral elements (selenium 102 ug/L and zinc (1145 ug/L), glucose (79 mg/dL), lipid (Total cholesterol: 127 mg/dL; HDL 52 mg/dL, LDL: 56 mg/dL; triglycerides: 97 mg/dL), protein (7.4 g/dL). No alterations were observed in bone (total calcium: 9 mg/dL; phosphate: 3.3 mg/dL) or thyroid metabolism (TSH: 0.95 µUI/mL; free T4: 1.06 ng/dL). Patient's hemoglobin levels (11.8 g/dL) along with normocytosis (mean corpuscular volume: MCV=92.2 fL) and normal status of serum iron (68 µg/ml), transferrin (259 mg/dL), total capacity of iron transport (365 µg/ml), transferrin saturation index (19%), B12 vitamin (491 pg/mL) and serum folate (17.3 pg/mL) ruled out anemia. Vitamins A and C where low but within the reference range at baseline. Of note, decreased serum prealbumin levels and 25-hydroxy-vitamin D deficiency, indicative of mild malnutrition at baseline, were normalized after the second ADMSC infusion for up to 6 months, along with a 3 kg weight gain (Supplementary Tables 3 and 4, Figure 3 B).

Table S5- Summary of selected items scored by BEBSS

Item Scored	PRE-TREATMENT	21 d	42 d	2 mo	3 mo	6 mo	9 mo	1 yr	2 yr
Area (Max. 50)	11.5	11.5	11.5	11	6.25	6	6.75	13.5	14.75
Mouth (Max. 5)	5	5	5	5	5	5	2	2	5
Eyes (Max. 5)	1	1	1	1	1	1	0	0	1
Larynx (Max. 5)	1	1	1	1	1	1	0	0	0
Esophagus (Max. 5)	5	5	5	5	5	5	3	3	2
Nutricional compromise (Max. 5)	3	3	3	3	3	3	2	2	2
**Total score (Max. 100)**	40.13	40.13	40.13	39.63	34.88	34.63	27.38	34.13	35.38

BEBSS: Birmingham Epidermolysis Bullosa Severity score. d: days after first infusion; mo: months after first infusion; yr: years after first infusion.

Table S6- Summary of selected items scored by EBDASI

EBDASI DISEASE ACTIVITY	PRE-TREATMENT	21 d	42 d	2 mo	3 mo	6 mo	9 mo	1 yr	2 yr
Skin (Max. 120)	35	33	27	13	5	6	8	11	13
Scalp (Max. 10)	0	0	0	0	0	0	0	0	9
Mucous membranes (Max. 120)	20	20	15	15	12	11	10	10	4
Nails (Max. 20)	0	0	0	0	0	0	0	0	0
Others epihelialized surfaces (Max. 6)	4	4	2	2	2	0	0	0	0
Total activity (Max 276)	59	57	44	30	19	17	18	21	17
**EBDASI DISEASE DAMAGE**	**PRE-TREATMENT**	**21 d**	**42 d**	**2 mo**	**3 mo**	**6 mo**	**9 mo**	**1 yr**	**2 yr**
Skin (Max. 84)	35	33	35	34	30	30	37	34	42
Scalp (Max. 20)	1	1	1	1	1	1	1	1	1
Mucous membranes (Max. 120)	8	8	8	8	8	8	8	8	8
Nails (Max. 60)	54	54	54	54	54	54	54	54	54
Others epihelialized surfaces (Max. 50)	16	16	10	16	16	16	16	16	16
Total damage (Max. 230)	114	112	114	113	109	113	116	113	121
Total score (Max. 506)	173	169	158	143	128	130	134	134	138

EBDASI: Epidermolysis Bullosa Disease Activity and Scarring Index d: days after first infusion; mo: months after first infusion; yr: years after first infusion.

Table S7- Fluorochrome-conjugated antibodies for flow cytometry analysis

Antibody (anti human-)	Fluorochrome	Vendor/Cat. No./Clone
CD3	FITC	Biolegend/ #300306/ HIT3a
CD4	PercP-Cy5.5	BioLegend/ #317428/ OKT4
CD4	PE	BioLegend/ #317410/ OKT4
CD8	PercP-Cy5.5	BioLegend/ #344710/ SK1
CD14	APC	Immunostep / #14A 100T/47-3D6
CD15	FITC	BioLegend/ #301904/ HI98
CD16	FITC	Mylteny/ #130-091-244/ VEP13
CD25	APC	BioLegend/ #302610/ BC96
CD33	PeP-Cy5.5	BD Biosciences/ #551377/ WM-53
CD45RA	APC	BioLegend/ #304112/ HI100
CD45RO	PE	BioLegend/ #304206/ UCHT1
CD56	PE	Mylteny/ #130-100-653/ AF12-7H3
FOXP3	PercP-Cy5.5	eBiosciences/ #45-4776-42/ PCH101

Table S8- Flow Cytometer setup parameters

Instrument: FACSCalibur (BD Biosciences)					
Laser lines	488 nm. Argon-ion 15mW			635 nm. Red diode	Laser lines
Emission filters	FL1 530/30	FL2 585/42	FL3 650LP	FL4 661/16	Emission filters
Fluorochrome	FITC	PE	PercP-Cy5.5 PeP-Cy5.5	APC	Fluorochrome
